# Management of Calcified Canals with a New Type of Endodontic Static Guide: A Case Report

**DOI:** 10.3390/dj12060166

**Published:** 2024-06-03

**Authors:** Roberto Fornara, Massimo Pisano, Giovanni Salvati, Giulia Malvicini, Alfredo Iandolo, Carlo Gaeta

**Affiliations:** 1 Independent Researcher, 20010 Milan, Italy; rfornara@unisa.it; 2 Department of Medicine, Surgery and Dentistry “Scuola Medica Salernitana”, University of Salerno, 84081 Baronissi, Italyaiandolo@unisa.it (A.I.); 3 Unit of Endodontics and Restorative Dentistry, Department of Medical Biotechnologies, University of Siena, 53100 Siena, Italy; g.salvati2@student.unisi.it (G.S.); c.gaeta@student.unisi.it (C.G.)

**Keywords:** guided endodontics, pulp canal obliteration, apical periodontitis, access cavity, cone beam computed tomography, intraoral scanning

## Abstract

(1) Background: Pulp canal obliteration (PCO) is a common condition characterized by an abnormal dentinal apposition within the canal wall, which often rises as a consequence of dental trauma. In recent years, “guided endodontics” has offered a reliable and safer procedure for endodontic access in case of PCO. The present case report aims to introduce a new endodontic guided system with a sleeveless, open-frame titanium guide designed to reduce costs compared to traditional templates. (2) Methods: The patient is a 19-year-old female who was referred to a private clinic to treat a calcified and symptomatic tooth (3.3) with apical periodontitis. Following the first unsuccessful treatment attempt using the operating microscope, a three-dimensional cone beam computed tomography (CBCT) examination and an optical impression were performed in agreement with the patient. The Digital Imaging and Communications in Medicine (DICOM) and Standard Tessellation Language (STL) files were sent to the lab for the template packaging, and the patient was scheduled for a second appointment. The endodontic guide was accurately positioned, and shaping and filling were concluded with success. The canal was sealed with the single-cone technique and bioceramic sealer. (3) Results: The patient reported no significant post-operative symptoms. Notably, the treatment achieved total symptom resolution, as evidenced by radiographic assessments conducted 3 and 24 months post-treatment, confirming the procedure’s success. (4) Conclusions: This innovative sleeveless, open-frame static titanium guide presents a promising advancement in guided endodontics for PCO treatment. The advantages include preserving healthy dental tissue, reduced chairside operating time, and cost savings compared to conventional templates. This approach holds promise for enhancing the quality and efficiency of endodontic procedures in cases of PCO. While the static guide holds promise, larger prospective studies are necessary to validate its efficacy, safety, and broader applicability in routine endodontic procedures.

## 1. Introduction

Pulp canal obliteration (PCO) or calcific metamorphosis (CM) is defined as the deposition of hard tissue within the pulp chamber mainly attributed to trauma [1], most often found in teeth with concussion and luxation injuries [2]. The exact mechanisms of calcific tissue deposition leading to PCO remain largely unknown [2]. However, theories suggest factors like neurovascular damage, sympathetic nervous response, regulatory changes in odontoblasts, and blood clot-induced calcification as potential causes [2].

PCO is frequently discovered by chance and presents a clinical challenge. Its nature is often asymptomatic [2], prompting clinicians to decide whether to initiate root canal treatment [3].

Treatment is recommended in the presence of clinical symptoms such as periapical lesions, dyschromia [2,4], tenderness to percussion with a PAI score ≥ 3, and a negative response to sensibility testing [2]. The American Association of Endodontics (AAE) categorizes PCO cases as moderate to high risk [5], due to the potential treatment failure, increased iatrogenic errors, and excessive dentine removal [6]. The primary objective in treating PCO is to access the canal system conservatively, minimizing errors such as false paths and/or perforations [1]. The use of the dental operating microscope, long shank burs, and ultrasonic instruments has enhanced the predictability of calcified canal negotiation [2]. A recent study reported that the operating microscope enables the detection of all root canals, establishing full working length in 90% of the cases, with an 80% success rate observed after a 3-year follow-up [7]. Despite these promising results, the process of canal negotiation is extremely time-consuming and reliant on the patient’s cooperation [7]. Furthermore, successful negotiation of an obliterated root canal may result in significant loss of hard tissue, particularly in the cervical region, which might impair stability and reduce the long-term prognosis of the tooth [8].

To increase the success rates of endodontic treatment of calcified canals, “guided endodontics” has been developed as an alternative to traditional treatments [3]. This technique is performed using a static template or through dynamic navigation. Static guidance involves the use of a template that guides the drill during the clinical procedure to reach the “target point” [3]. On the contrary, dynamic navigation is based on markers located in the patient’s mouth and a real-time camera system that guides the clinical procedure [9,10,11]. The traditional workflow of guided endodontics derives from the principles of guided surgery for implant placement [3] and, recently, this technology has also been proposed for fiber post removal [10,11].

Endodontic access templates can have various shapes and characteristics and be packaged with different materials [3,12]. These 3D guide-based techniques require the design of patient-specific templates for canal negotiation using cone beam computed tomography (CBCT) of the affected tooth [3]. The CBCT data [The Digital Imaging and Communications in Medicine (DICOM)] and those from the optical impression [Standard Tessellation Language (STL)] are combined in virtual imaging software. This software overlays the planned negotiation path onto scanned images of the tooth to create an access opening template that will guide the drill during the clinical procedure to reach the target point [13]. In the operative phase, the access will be guided by the presence of the endodontic guide, reducing the risks of the freehand technique [3,14,15].

Recently, a few groups of researchers have introduced and tested a novel open-frame and sleeveless static surgical titanium guide for implant placement [16]. These studies have demonstrated that this guided surgery system is accurate in implant dentistry [16]. Despite its potential benefits in the field of endodontics, this open-frame and sleeveless guide has never been implemented in endodontic practice.

This case report aims to introduce and explore the application of a new endodontic-guided system with a sleeveless, open-frame titanium guide to treat a PCO tooth with acute symptoms and apical periodontitis, laying the groundwork for future validation through more extensive research.

## 2. Case Report

A 19-year-old female patient was referred to a private clinic to treat a calcified and symptomatic tooth 3.3. She had no history of dental trauma and was in good general health, with her only dental history being a past fixed orthodontic treatment.

On clinical examination, tooth 3.3 negatively responded to cold and a percussion test with no pathological probing pocket depth. The patient reported spontaneous nocturnal pain, which persisted despite the use of non-steroidal anti-inflammatory drugs.

**Figure 1:**
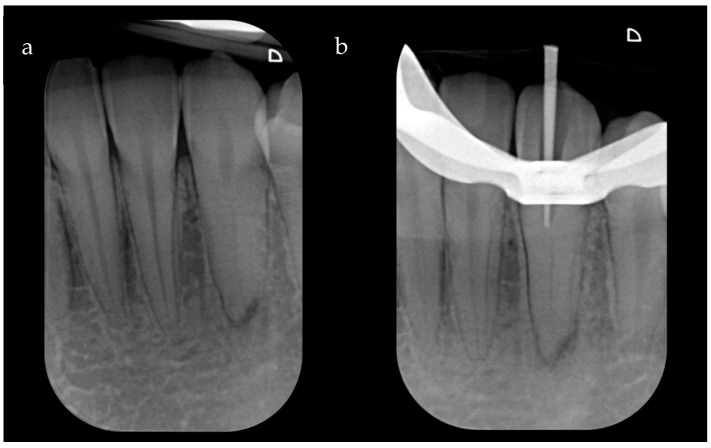
(**a**) Pre-operative X-ray; small periapical radiolucent area. (**b**) Intra-operative X-ray with a gutta-percha cone to evaluate the trajectory of the initial preparation carried out with ultrasonic inserts. It is impossible to reach the canal despite using the operating microscope, and the cone shows that one is proceeding in the wrong direction.

An initial periapical X-ray using the long-cone parallel technique revealed a clearly identifiable root canal space and periapical radiolucency of PAI 3 (changes in bone structure with light mineral loss) (Figure 1a). Tooth 3.3 was diagnosed as necrotic pulp with symptomatic apical periodontitis and was planned for nonsurgical root canal therapy. Considering the pre-operative, X-ray which notably showcased the root canal, a pre-operative CBCT was deemed unnecessary, and it was decided to proceed with the traditional treatment consisting of the freehand ultrasonic technique and a dental operating microscope.

After local anesthesia (Articaine Septanest with adrenaline 1:200,000; Septodont, Saint-Maur-des-Fosses, France), and rubber dam isolation, the access cavity was prepared under a dental operating microscope (Leica M500, Leica Microsystems, Wetzlar, Germany) with a diamond access bur (Dentsply Sirona 851012 FG safe end bur, Dentsply Sirona, Charlotte, NC, USA). Afterwards, an ultrasonic handpiece (Satelec, Acteon, Merignac, France) with an Endodontic Treatment Exploration Ball Diamond Tip ultrasonic tip (Satelec P5^®^ Piezo, Acteon, Merignac, France) was used in endo mode. However, this initial attempt to negotiate the canal led to a small deviation that hindered further progress. Indeed, an intra-operative X-ray with a gutta-percha cone was performed, which showcased a small deviation that was hindering further progress (Figure 1b). Given the expressed desire of the patient to preserve the natural element, a different treatment strategy was proposed.

**Figure 2:**
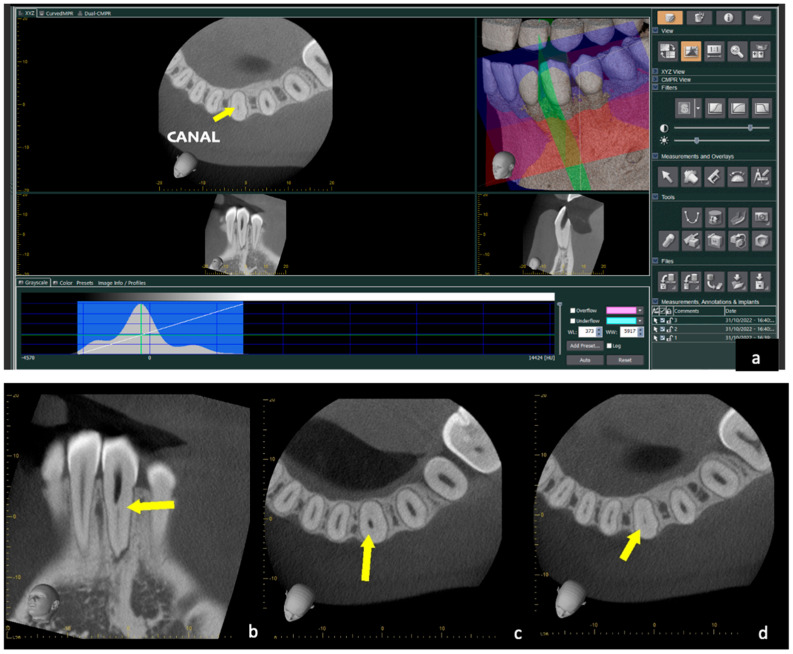
(**a**) Acquisition of 4 × 4 CBCT volume: the yellow arrow in the axial section shows the canal, while the created step is highlighted in the sagittal section. (**b**) Sagittal section of CBCT: the yellow arrow indicates the canal obstruction and the step created in the first attempt to locate the canal. (**c**) Axial section of CBCT: the yellow arrow indicates deviation from the canal. Three-dimensional simulation of the drill’s path in relation to the bio-model and the template. (**d**) Axial section of the most apical CBCT where the yellow arrow indicates the root canal.

**Figure 3:**
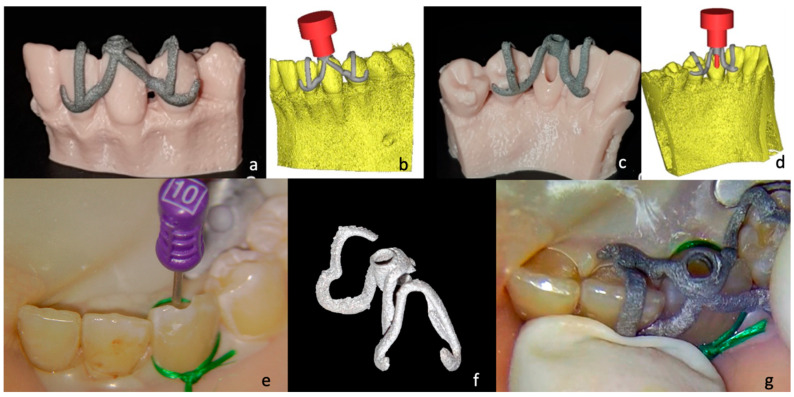
(**a**) Titanium template packaged and placed on the 3D-printed model: buccal view, (**c**) lingual view. (**b**) Three-dimensional simulation of the drill’s path in relation to the bio-model and the template: buccal view, (**d**) lingual view. (**e**) Intra-operative image of the K-file was inserted immediately after locating the canal. (**f**) The printed titanium template: the access cylinder can be customized entirely (in diameter and height). (**g**) Intra-operative image with the template correctly positioned.

The patient was informed of the anatomical complexity and consented to perform a pre-operative CBCT with a small FOV (Morita Veraview X800, J.Morita, Tokyo, Japan) and high resolution. Before proceeding, written informed consent was given by the patient after reading a patient information sheet. The selected FOV was the lowest available (6 × 7 cm in the lower jaw), using the advanced function (160-micron voxel) and then reconstructing the raw data with 80-micron voxels. The CBCT confirmed a zone of obstruction and a ledge created during the first attempt to negotiate the canal, in the mid/coronal third, and a buccal/lingual curvature (Figure 2 and Figure 3).

In agreement with the patient, an individual static-type endodontic guide was decided upon. In order to proceed, an optical impression was taken using an intraoral scanner (Aadva, IOS 100P, GC, Leuven, Belgium) to obtain a virtual model of the patient’s arch.

DICOM CBCT files and STL data from optical impressions (Carestream, Rochester, NY, USA) were matched and sent to the dental laboratory. Special medical software (Mimics—Materialise, Leuven, Belgium) was used to process the data and was validated by automatic segmentation management by artificial intelligence.

### Template Production

The STL file of the optical intraoral scan and the DICOM data of the CBCT were exported in a specific software (Geomatic) for the definition of the target. A template was modeled as a skeleton of a removable partial prosthesis, printed in titanium using an additive manufacturing process with an SLM (Selective Laser Melting My Sint100 Sisma) with Grade 23 medical titanium. When the template was printed, the entire build platform was thermally treated in an oven (GLOW, Mihm-Vogt, Stutensee-Blankenloch, Germany) for heat relaxation. The 3D model of the patient derived from the optical impression was printed with a professional DLP printer (SOL LCD printer Ackuretta, Taipei City, Taiwan) in epoxy resin (ADORPRINT, Ador, Hilden, Germany). The 3D model was used to finalize the template (Figure 3). Once the template was made, the patient was scheduled for a second appointment.

**Figure 4:**
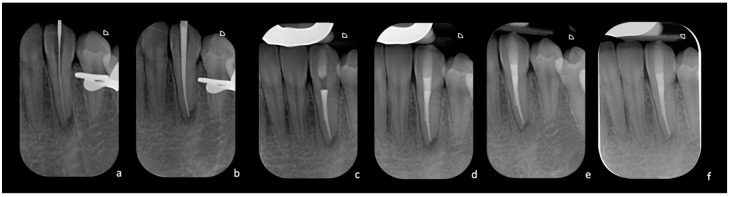
(**a**) Intra-operative X-ray of the K-file inserted at WL after locating the canal. (**b**) Intra-operative X-ray with the gutta-percha cone test. (**c**) Post-operative X-ray with completed root canal filling. (**d**) Radiograph taken after completing the coronal seal. (**e**) Radiographic control at three months with signs of improvement of the apical radiolucency. (**f**) Radiographic follow-up at two years with complete healing.

After local anesthesia (Articaine Septanest with adrenaline 1:200,000; Septodont, Saint-Maur-des-Fosses, France), the operating field was isolated with a rubber dam. The correct positioning and stability of the guide were preliminary checked, and the temporary filling material was removed. Once the template was positioned, a 0.8 mm drill (210L16 205 008 Komet Dental Gear Brassier GmbH & Co. KG, Lemgo, Germany) was mounted on the blue ring contra-angle and inserted through the guide cylinder. Once the planned target point was reached, the scouting phase was performed by a K-File #10 instrument (Dentsply Sirona Endodontics, Ballaigues, Switzerland) (Figure 3). The electronic working length was measured using the electronic apex locator (Ai-Pex, Gullin WoodPecker Medical Instrument Co., Ltd., Guilin, China), and a periapical X-ray was performed to confirm the working length. The canal was shaped with rotary Ni-TI files (EdgeEndo Platinum, EdgeEndo, Albuquerque, NM, USA), and the apex was finished with an F3 file (Protaper Ultimate, Dentsply Sirona, Charlotte, NC, USA). Detersion was performed by multiple rinses of 5% sodium hypochlorite (Niclor 5—Ogna, Muggio dental division, MB—Italy). To seal the shaped canal, the single-cone technique with hydraulic cement (Endosequence BC Sealer, Brasseler USA Savannah, GA, USA) was used; the cavity was then provisionally sealed with temporary cement. After four days, the patient did not show any sign of inflammation and symptoms, and restoration was then performed with adhesive (Optioned Solo^TM^ Plus, Kerr Corporation, Orange, CA, USA) and a composite technique (Enamel plus, HFO Micerium, Rosbach, Germany). A final radiograph was performed at the end of the restorative procedures (Figure 4).

## 3. Discussion

The treatment of symptomatic apical periodontitis in the case of PCO poses a significant challenge for endodontists [3]. The potential for iatrogenic errors and excessive dentin removal can adversely impact the prognosis of endodontically treated teeth [13]. However, guided endodontics seems to be a new and safe approach to this clinical situation do [17].

This case report introduces a pioneering guided endodontic approach for PCO in a lower left canine with apical periodontitis. This is the first case report that attempts to apply a guided system with a sleeveless open-frame titanium structure in endodontics, recently introduced in implantology [16]. Before the advent of guided endodontics, managing PCO depended largely on clinical expertise [2]. The non-guided technique for negotiating obliterated canals involved the dental operating microscope, long shank burs, ultrasonic instruments, and multiple radiographs to confirm and adjust the negotiation path [2]. While effective, this approach is time-intensive, relies heavily on patient cooperation [7], and poses risks of instrument separation, perforation, and excessive dentin removal [1]. Yet, many of these issues can be addressed with the application of guided endodontics [3].

In the present case report, the patient’s history of fixed orthodontic treatment is noteworthy, potentially contributing to PCO, as demonstrated in the literature [4]. The pre-operatory X-ray clearly revealed the root canal, giving no indication of potential complications. However, despite these expectations, our initial attempt to treat the canal with ultrasounds and a microscope led to a small deviation that hindered further progress. The decision to use an intra-operatory guide was essential to safeguard the integrity of the tooth while navigating this complex procedure. The purpose of guided endodontics is in fact to reduce treatment time and minimize the risk of technical error [18,19]. In this case, a new static guide with a smaller size was employed, enhancing visibility and reducing the risk of saliva contamination under the rubber dam. The design of the static guide improved control during the procedure, offering better irrigation supply to the root canal system and greater temperature control during the drilling phase [16]. Furthermore, to prevent unnecessary wear of the tooth structure, a drill of 0.8 mm was used. This choice was prompted to address concerns from previous studies that reported high wear of dentin due to an excessive bur diameter [20,21,22]. Moreover, in a few minutes, patency was achieved, reducing the chair time required to negotiate the canal with the traditional method. Without the help of guidance, even the most experienced operators should be careful and take multiple radiographs to verify the accurate position of the bur [2]. Indeed, guided endodontics reduces the need for numerous radiographs, counterbalancing the radiation exposure associated with the CBCT [16,22]. Overall, the clinical results of the present case report have been gratifying.

Several advantages have been encountered while using this innovative system in guided endodontics. First of all, the system is customizable and allows us to eliminate the sleeves which are present in the vast majority of guided endodontic and implant surgery systems on the market today. Indeed, the sleeves are useful to guide the burs but force the clinicians to use dedicated surgical kits with long drills due to the large amount of space they occupy. In addition, the open template allows us to check each supporting tooth within the section, enabling precise assessment of the guide’s fit. Furthermore, the choice to use a titanium-based guide instead of a resin-based guide derives from the fact that other groups of researchers were using the same guide for implant placement with promising results [3]. Compared to metal guides, resin guides are cheaper and easier to print and they can be easily adapted to the site in the case of minimal misfits [16]. However, when working with resin guides, it is essential to avoid delays in the treatment because the stability of these guides over time is not comparable to that of metal guides [16]. Therefore, the strengths of the new static guide lie in its cost-effectiveness, the use of readily available drills, greater visibility and irrigation, remarkable stability, and the potential to lower the CBCT FOV volume, thereby increasing the quality of the images.

However, it is essential to highlight that a significant limitation of static guidance lies in its restriction to straight roots or the straight portions of curved roots. This limitation is a crucial consideration, as the technique may not be suitable for navigating the complexities of more intricately curved root structures [3]. Nonetheless, static guidance becomes feasible when the target point is situated before the curvature. In any case, deeper localization of the canal calcification could affect the precision of the template by increasing the bending of the bur, as recently described [14]. Furthermore, only a limited number of clinicians routinely use guided endodontics today, due to the high cost of the required machines (CBCT, intraoral scanners, and 3D printers), the time required for the planning process, and the necessary learning curve for the clinician to learn how to design with software [3].

While the present case report demonstrates the successful application of a new static guide for guided endodontics in the treatment of PCO, it is important to acknowledge certain limitations inherent in these data. Firstly, the case is singular, and the findings may not be fully representative of the broader population with PCO. A larger sample size and a comparative analysis with traditional treatment approaches would provide a more robust understanding of the efficacy and safety of the presented static guide. Finally, the use of less experienced operators in the application of the static guide was not specifically addressed in this case report. Future investigations should assess the learning curve associated with this technique and evaluate its feasibility among operators with varying levels of expertise.

In conclusion, while this case report highlights the potential benefits of the new static guide in guided endodontics for PCO, these limitations underscore the necessity for further prospective clinical studies.

## 4. Conclusions

This innovative sleeveless, open-frame static titanium guide presents a promising advancement in guided endodontics for PCO treatment. The advantages include preserving healthy dental tissue, reduced chairside operating time, and cost savings compared to conventional templates. This approach holds promise for enhancing the quality and efficiency of endodontic procedures in cases of PCO. While the static guide holds promise, larger prospective studies are necessary to validate its efficacy, safety, and broader applicability in routine endodontic procedures.

## Data Availability

The data presented in this study are available on request from the corresponding author.
